# An integrated landscape of protein expression in human cancer

**DOI:** 10.1038/s41597-021-00890-2

**Published:** 2021-04-23

**Authors:** Andrew F. Jarnuczak, Hanna Najgebauer, Mitra Barzine, Deepti J. Kundu, Fatemeh Ghavidel, Yasset Perez-Riverol, Irene Papatheodorou, Alvis Brazma, Juan Antonio Vizcaíno

**Affiliations:** 1 European Molecular Biology Laboratory, EMBL-European Bioinformatics Institute (EMBL-EBI), Hinxton, Cambridge, CB10 1SD UK; 2 Department of Informatics, University of Bergen, 5020 Bergen, Norway

**Keywords:** Cancer models, Data integration, Proteome informatics

## Abstract

Using 11 proteomics datasets, mostly available through the PRIDE database, we assembled a reference expression map for 191 cancer cell lines and 246 clinical tumour samples, across 13 lineages. We found unique peptides identified only in tumour samples despite a much higher coverage in cell lines. These were mainly mapped to proteins related to regulation of signalling receptor activity. Correlations between baseline expression in cell lines and tumours were calculated. We found these to be highly similar across all samples with most similarity found within a given sample type. Integration of proteomics and transcriptomics data showed median correlation across cell lines to be 0.58 (range between 0.43 and 0.66). Additionally, in agreement with previous studies, variation in mRNA levels was often a poor predictor of changes in protein abundance. To our knowledge, this work constitutes the first meta-analysis focusing on cancer-related public proteomics datasets. We therefore also highlight shortcomings and limitations of such studies. All data is available through PRIDE dataset identifier PXD013455 and in Expression Atlas.

## Introduction

Cancer cell lines are powerful models often used in place of primary cells to study for instance the molecular mechanisms of the disease. Cell lines derived from patients provide an inexpensive source of pure cell populations and are easy to manipulate and characterize. Although they usually retain driver mutations, cell lines often contain ‘additional’ genomic aberrations not present in tumours. They also lack the tumour microenvironment interactions and can undergo divergent evolution during long-term cell culture^1–3^. Hence, in order to capture the patient’s tumour biology, it is therefore often more desirable to study primary cells or tissue biopsies.

Thanks to a number of International initiatives and large-scale studies, a variety of omics approaches have been employed to characterise both tumour samples and cell lines at the molecular level. Among them, The Cancer Genome Atlas (TCGA) used next-generation sequencing (NGS) and reverse-phase protein arrays (RPPAs) to generate genome and expression landscapes in approximately 10,000 tumour specimens^4^, and contains over 20,000 samples in total. The Cancer Cell Line Encyclopedia (CCLE) consortium measured DNA copy-number, mutations and gene expression in 1,072 human cancer cell lines^5^. Also in this context, the Genomics of Drug Sensitivity in Cancer (GDSC) project provided genomic and gene expression data for over 1,000 cell lines combined with cell line sensitivity measurements related to a wide range of anti-cancer therapeutics^6^.

While the majority of the available data is based on NGS technologies measuring DNA and RNA-level alterations in cancer, proteins are however most often the functional molecules, providing a link between genotype and the phenotype. Proteins are also the targets of many drugs and can be a source of novel biomarkers. Mass spectrometry (MS) is the main proteomics technology, capable of providing system-wide measurements of protein expression, post-translational modifications and/or protein-protein interactions, among other pieces of information^7,8^. Accordingly, cancer cell lines proteomes have been characterised in a number of MS-based studies^9–17^. However, due to technological limitations, these and other currently publicly available proteomics datasets are usually smaller in scope in comparison to analogous genomics and transcriptomics studies. Therefore, although they can routinely cover thousands of gene products, the datasets are usually limited to tens of samples. MS-based protein expression information in tumour specimens has also been obtained, for example through the work of the Clinical Proteomic Tumour Analysis Consortium (CPTAC)^18,19^ or by other independent groups^20–22^. Additionally, RPPA approaches can be used to characterise a usually much smaller number of proteins^5,23^.

Importantly, the MS data underpinning these efforts is now routinely made freely available in the public domain, an opposite situation to the state-of-the-art just a few years ago. Particularly, the PRIDE database^24^ is the world-leading resource as part of the ProteomeXchange Consortium, storing raw files, processed results and the related metadata coming from thousands of original datasets. Public datasets stored in PRIDE, together with existing ones in other open proteomics repositories (e.g. the CPTAC portal^25^ or MassIVE^26^) present an opportunity to be systematically reanalysed and integrated, in order to confirm the original results, potentially obtain new insights and be able to answer biologically relevant questions orthogonal to those posed in the original studies. Such integrative meta-analyses have already been successfully employed in different omics data types, especially in genomics and transcriptomics^27^. For example, Lukk *et al*.^28^ integrated thousands of microarray files to compile a map of human gene expression. In metabolomics, Reznik *et al*.^29^ used MS data from eleven studies to measure the extent of metabolic variation across tumours. A similar trend is starting to be observed in proteomics, where reuse of public datasets is becoming increasingly popular, with multiple applications^30,31^. Some examples where joint reanalysis of large public datasets has been performed involved the creation of comprehensive maps of the human proteome^32^ and of human protein complexes^33^, or the characterisation of the functional human phosphoproteome^34^.

However, to our knowledge no previous studies have attempted to reanalyse and integrate quantitative proteomics datasets in order to provide a global reference map of protein expression in cancer. Here, we provide a reference resource of protein expression across different types of primary tumours and the corresponding cell line models (246 clinical tumour samples and 191 cancer cell lines), using public proteomics datasets as the base.

## Results

### A catalogue of cancer cell lines and primary tumour proteomes

**Fig. 1:**
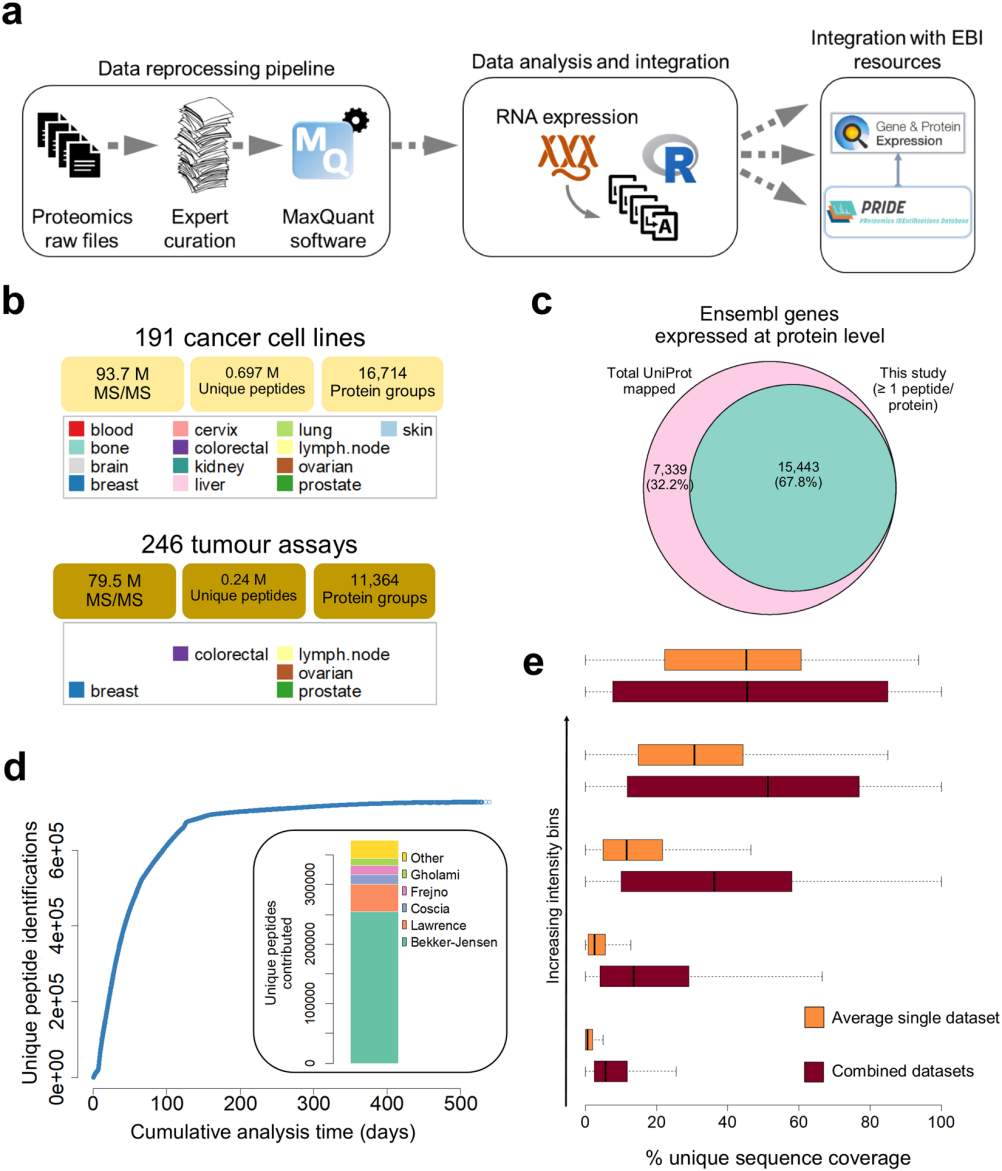
(**a**) Overview of the study design and the data reanalysis pipeline. (**b**) Summary of the total number of MS/MS spectra, number of unique peptides and protein groups identified in the cell line and tumour datasets. (**c**) Overlap between Ensembl protein-coding genes identified and all theoretical genes annotated in UniProt. Only protein groups identified by at least one unique peptide were included. (**d**) Plot showing the cumulative MS analysis time versus the cumulative number of unique peptide identifications obtained. Inset: barplot showing the proportion of peptides uniquely identified in each individual dataset. (**e**) Distribution of protein sequence coverage in the cell line data, stratified into bins of increasing protein intensity, in the combined dataset (brown boxplots) and an average calculated across 7 individual datasets (orange boxplots).

We selected, manually curated and re-annotated 7,171 MS runs coming from 11 large-scale quantitative cancer related proteomics studies (Online-only Table 1) (Fig. 1a). We restricted the studies to those where protein quantification was based on the intensity of the peptide precursor ions (MS-1 based quantification) and the studies employed the same MS platform (Thermo Orbitrap). The combined analysis yielded an aggregated dataset of protein expression in 191 cancer cell lines and 246 clinical tumour samples (Fig. 1a). In addition, 35 non-malignant tissues, present in the original publications, were also included in the combined dataset. Cell line samples originating from 13 different tissue-origins were included: blood, bone, brain, breast, cervix, colorectal/large intestine, kidney, liver, lung, lymph node, ovarian, prostate and skin. The patient-derived samples came from breast, colorectal, ovarian and prostate tumours, and from breast to lymph node metastases. Lineage annotation of the cell lines and tumour samples is included in Supplementary Table 1. Overall, over 173 M spectra were reanalysed using MaxQuant (MQ)^35^. The resulting aggregated dataset covered 15,443 gene products with at least one unique (unambiguous) peptide evidence, which corresponded to a 67.8% coverage of the entire UniProt reference human proteome (Fig. 1c). Since quantitative proteomics data originating from different studies can be heterogeneous and likely to contain batch effects, we developed and benchmarked a procedure to integrate appropriately the quantification data. Based on that, we obtained quantification values for an average of 6,593 proteins per cancer cell line and 5,371 proteins per tumour type.

From the information available in the raw files we inferred that it would take over 538 days of mass spectrometer time to repeat all of the original experiments (Fig. 1d), ascertaining the potentially huge benefit to performing the *in silico* data reanalysis. In addition, the aggregation of individual datasets increased the global proteome coverage. In this study, each dataset contributed was quite heterogeneous and ranged between 1,600 and 250,000 unique peptide identifications to the aggregated dataset (Fig. 1d inset), in parallel increasing the confidence and robustness in the protein identification and quantification analyses. This was particularly true for low abundance proteins, where the average protein sequence coverage in the aggregated dataset was typically higher than the sequence coverage found in individual datasets (Fig. 1e).

The overall results of the study have been made publicly available in two EMBL-EBI resources: Expression Atlas (EA)^36^ and the PRIDE database^24^. Expression Atlas provides the expression values for each dataset in a separate track (E-PROT-18 to E-PROT-28). PRIDE dataset identifier PXD013455 contains all the raw data, MQ intermediate files and the combined proteomics analysis results.

### Comparison of peptides detected in tumour and cell line samples

Our first objective was to use the peptides identified in the aggregated dataset to assess for differences in MS-detectable proteome, independent of the tissue of origin and/or the lineage, between tumour and cell line. On average, 6,208 proteins were detected in the majority of tumours of any given type (meaning in ≥50% of samples of that group) and 7,401 proteins in cell line data.

**Fig. 2:**
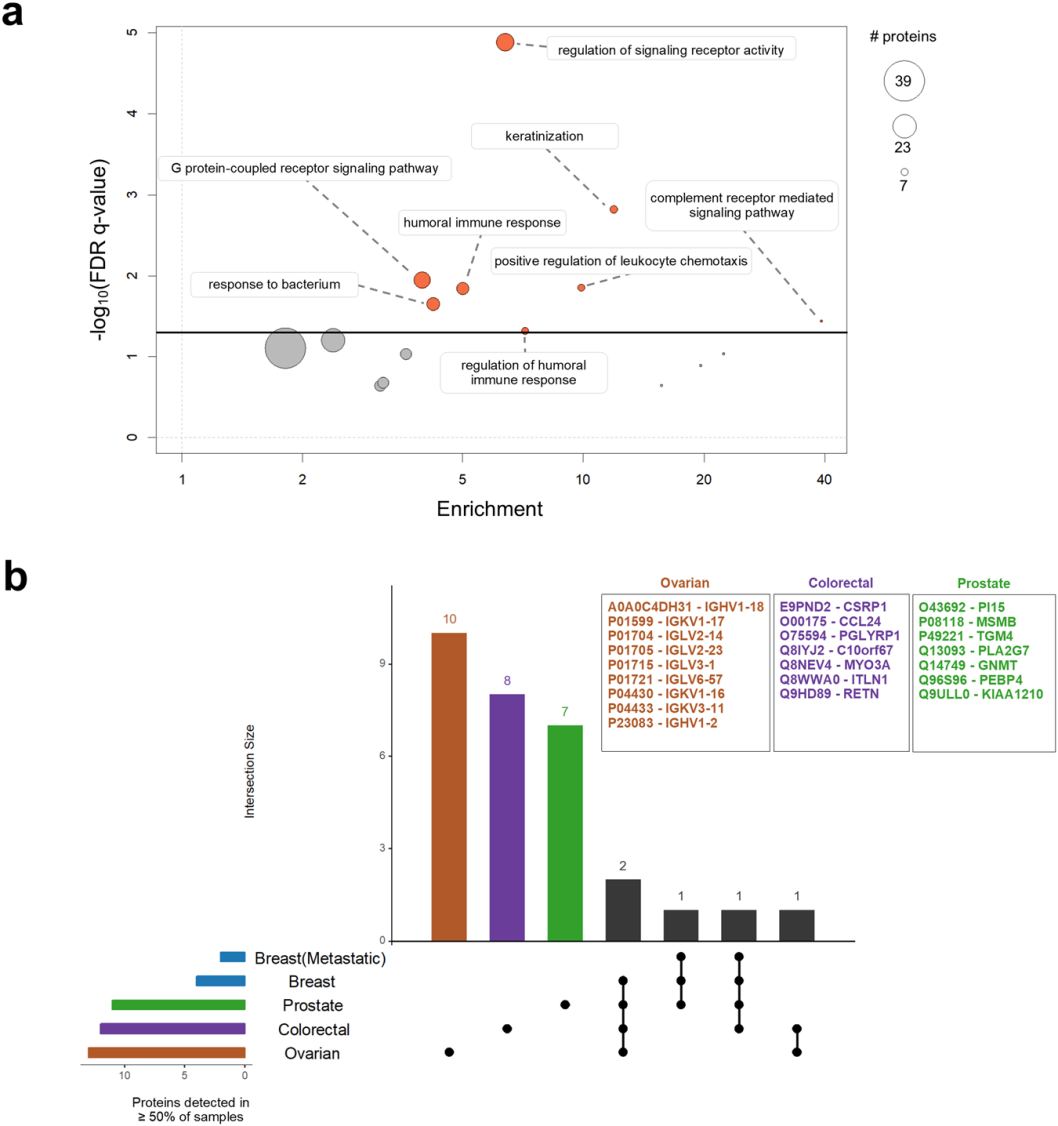
(**a**) Scatterplot summarising the enrichment analysis of biological process GO terms for the 300 proteins detected only in tumour samples. The x-axis represents the enrichment score, the y-axis represents the enrichment p-value corrected for multiple testing using the Benjamini and Hochberg method. The size of the circles corresponds to the number of proteins associated within a given category. (**b**) Counts out of the 330 tumour-specific proteins detected in the majority of samples (i.e. ≥ 50% of samples) across different tumour types. Colour coded horizontal bars show the total number of proteins detected in the majority of samples in each tumour type. Black circles indicate the intersections. Horizontal bars in the histogram show the number of proteins in the corresponding intersection. Proteins specific (as UniProt protein identifiers) to each tumour type are listed in the text boxes.

When we compared the peptides identified across all cancer cell lines versus all the tumours, we observed that only a small fraction was identified exclusively in tumour data. Out of 711,352 peptides, 33,045 (4.6%) were present only in tumours, constituting a *tumour-specific peptide set*. In contrast, a much larger proportion of peptides was identified only in cell lines (66.0%). This was expected as some of the cell line studies employed extensive fractionation protocols or used multiple digestion enzymes (for example, in dataset from ref. ^17^ the authors used four enzymes to obtain a deep proteome of HeLa, Online-only Table 1). From the *tumour-specific peptide set*, only peptides that uniquely matched to a protein sequence were retained, therefore enabling the unambiguous identification of the corresponding proteins. These 9,907 peptides mapped to 330 proteins for which no identification evidence was found in any of the cell lines. This is in our view an interesting finding given that the sequence coverage of the cell line datasets was much larger. Next, gene ontology (GO) enrichment analysis of this protein set was performed using GOrilla^37^ and REVIGO^38^, revealing that the tumour-specific proteins were most significantly enriched for biological processes associated with *regulation of signalling receptor activity* (GO:0010469). Other terms, significantly enriched at a FDR (False Discovery Rate) p-value < 0.05 level, included *keratinization* (GO:0031424), *G protein-coupled receptor signalling pathway* (GO:0007186), *positive regulation of leukocyte chemotaxis* (GO:0002690), *humoral immune response* (GO:0006959) and *response to bacterium* (GO:0009617) (Fig. 2). In addition, this protein set was enriched in *extracellular space* related cellular component GO terms, potentially suggesting that tumour-specific proteins could be involved in secretion. No tumour-specific proteins were consistently detected across all the tumour samples. However, ovarian, colorectal and prostate tumours showed lineage specific expression, as highlighted in Fig. 2b. After performing a pathway enrichment analysis using Reactome^39^ we found that the 10 proteins specific to the ovarian tumour samples were immunoglobulins enriched in elements of the *CD22 mediated BCR regulation pathway* (Reactome pathway identifier R-HSA-5690714, FDR p-value = 2.89 E-15).

Taken together, expression of proteins associated with those pathways is not detectable in the cell lines considered here. However, it is important to consider an inherent limitation in this type of studies. Tumour-unique peptides could have potentially originated from a number of non-tumour sources, for example tumour infiltrating immune cells (a different tissue) due to cross contamination during sample processing.

### Evaluating cell lines as tumour models based on protein expression correlation

In order to go beyond simple presence/absence qualitative measurements, we generated two matrices containing normalised protein expression measurements across cell lines and tumour samples.

We merged the two by cross-referencing the leading razor protein identifiers, which resulted in a union of 8291 protein profiles and an intersection of 4,476 proteins. These quantitative values, i.e. log2-transformed batch normalized ppb Intensities, were used to investigate the similarity between the samples.

Compared with the tumour tissues, cell lines showed similar levels of variability in protein expression. This was established using the coefficient of variation (CV) calculated between different cell lines and tumours within a given tumour type (i.e. reflecting cancer lineage sample-to-sample variability). The median CV was below 56% in all cases and in general, the majority of proteins. had CV values below 70% in both cell lines and tumours. Notable exceptions were ovary tumours and prostate cell lines which showed a much larger spread of CVs across individual proteins. Unsurprisingly, the variability between biological replicates (assessed in cell lines only) was lower than between samples from a different biological origin (median CV = 43%).

**Fig. 3:**
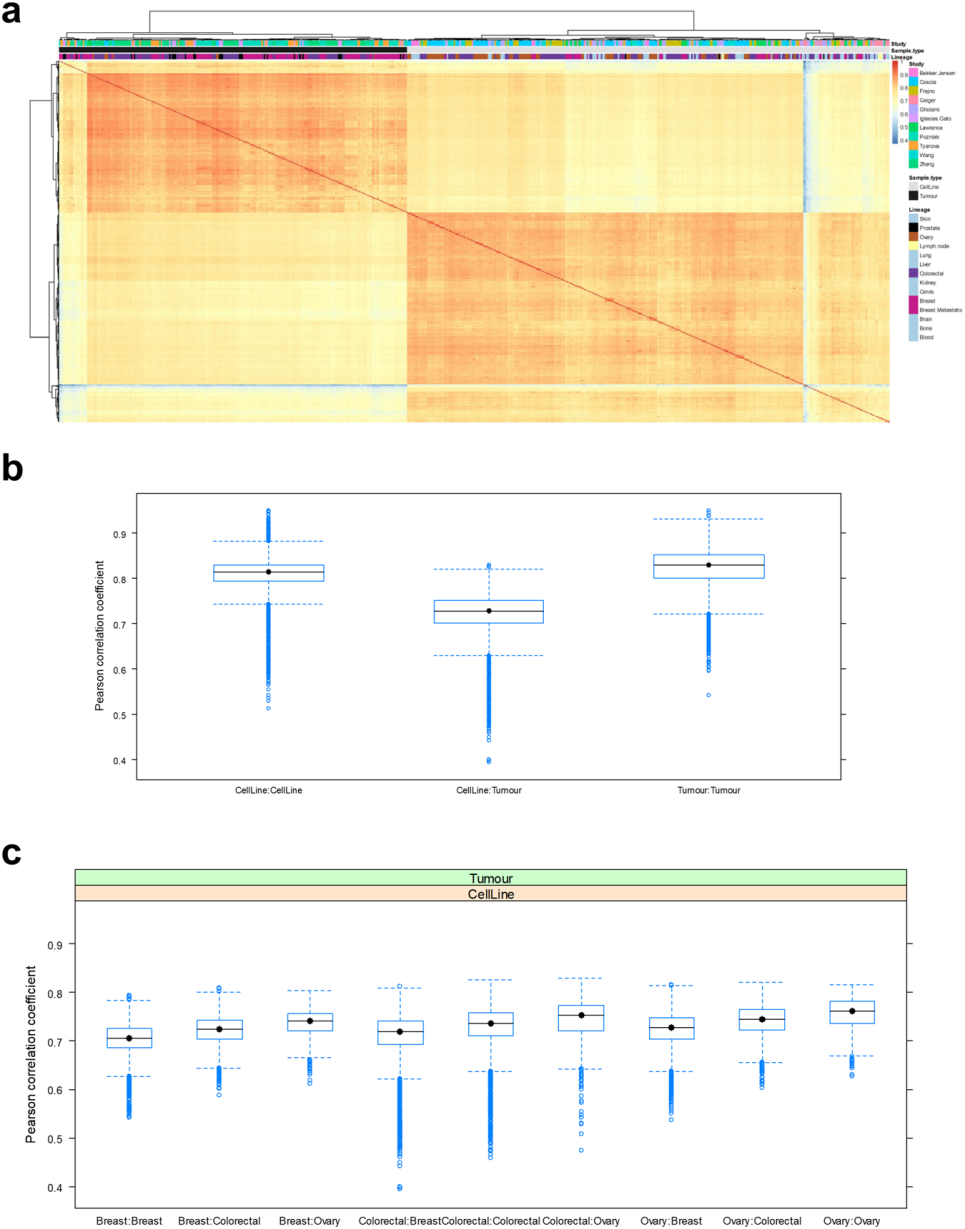
(**a**) Heatmap of the correlation coefficients across all samples. Colour corresponds to the r_p_, which was calculated using log2 transformed intensities and “pairwise.complete.obs” option. Hierarchical clustering using Euclidean distance was performed on both rows and columns. Boxplots showing the distribution of Pearson correlation coefficients corresponding to: (**b**) Correlations within and between sample types. The middle boxplot shows the correlation between cell lines and tumours; and (**c**) Distribution of correlation values between tumour samples and the different cell lines included within each tumour lineage.

To investigate the level of agreement in protein expression between endogenous tumour cells and their corresponding cell line models, non-parametric Pearson correlation coefficient (r_*p*_) values were calculated between all samples based on pairwise complete observations (Fig. 3a). We focused our analysis on three lineages with enough assays: colorectal, breast, and ovarian samples. Overall, molecular profiles appeared similar between all samples, as reflected by the relatively high r_*p*_ values, ranging from 0.39 to 0.95 and the median r_*p*_ of 0.77 (all correlations were significant with p-values « 0.01).

We found the similarities within sample types (either cell lines or tumours) to be higher than between sample types, as it might be expected. The median correlations within cell lines and tumours were similar: 0.81 and 0.83 respectively, whereas it was 0.73 between cell lines and tumours (Fig. 3b). Furthermore, even cell lines representing different cancer subtypes displayed a high correlation to all other samples. Interestingly, when examining correlations within specific lineages (i.e. tumours of one type to all cell line types) we found that cell lines from a corresponding lineage were not necessarily best correlated to the corresponding tumour type. For example, ovary cell lines showed best correlation to all tumour types (Fig. 3c). In the case of breast lineage, we found breast cell lines were the least correlated to breast tumours, including a wide distribution of those correlations. The corresponding calculations using Spearman correlation were also performed and showed similar results.

Overall, the analysis suggests that cell lines have similar baseline protein expression levels to those observed in tumours, as reflected by the high positive correlation found between all the samples. However, the existence of high correlations, even between different lineages, suggest that additional molecular features such as genomics alterations^40^ should be considered when selecting the most appropriate cancer model.

### Correlation between mRNA and protein expression in cancer cell lines

The correspondence between RNA and protein expression has been previously characterised in cell lines, tumour samples and tissues^41^. In most studies performed in tumour samples so far, the analysis was focused on single cancer types or otherwise, it was limited to a handful of samples for which both mRNA and protein measurements were available. By integrating the aggregated dataset with RNA-seq measurements publicly available already in EA (see Methods section), the correlation between mRNA and protein abundance was calculated for 134 cancer cell lines across 13 lineages, including 6,674 gene products that were overlapping between proteomics and transcriptomics data. The correlations were calculated for a total of 261 proteomics assays as some cell lines were measured in multiple studies/biological replicates.

### Within sample mRNA-protein expression correlation

**Fig. 4:**
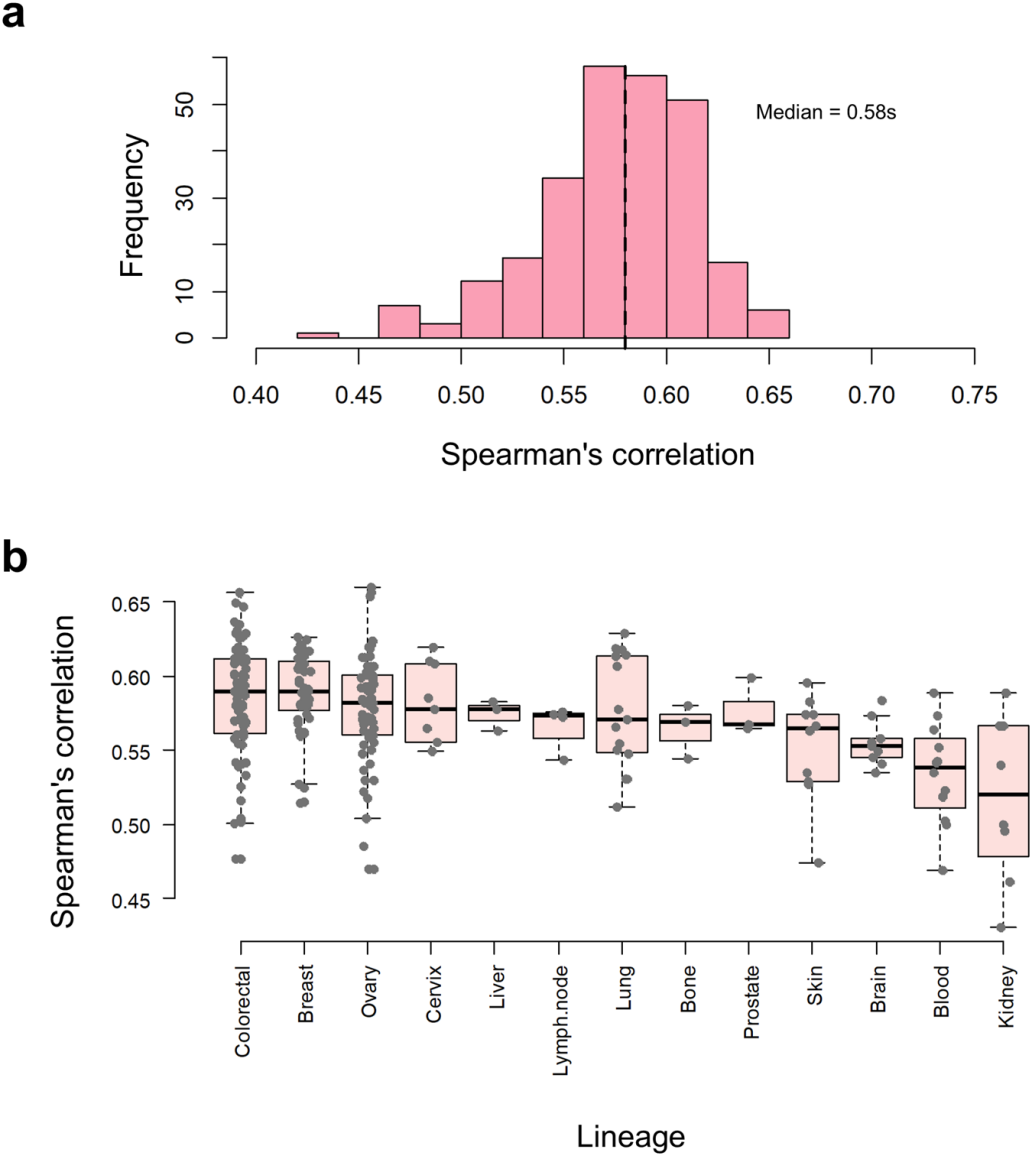
Correlation between mRNA and protein expression levels within the different cell lines. (**a**) Distribution of mRNA-protein Spearman correlation in 134 cancer cell lines (261 proteomics assys) originating from 13 lineages. The 134 cell lines were those common to this analysis and the existing RNA-seq data. (**b**) Boxplots showing the differences in distribution of mRNA-protein correlations between groups of cell lines originating from various lineages. The box plots show the median (horizontal line), interquartile range (box) and minimum to maximum values of the data. Only lineages with more than three cell lines were included.

To investigate the extent in which mRNA abundances are reflected at the protein level at steady state, the Spearman’s rank correlation coefficient (r_*s*_) was calculated for an average of 6,542 mRNA–protein pairs (some cell lines contained a higher number of mRNA-protein pairs than others), for each of the 261 proteomics assays (from 134 cell lines). Despite that the original omics measurements were performed in independent studies, all the cell lines displayed a statistically significant (p-value « 0.01) positive correlation. The median r_*s*_ was 0.58 and the values ranged between 0.43 and 0.66 (Fig. 4a). Box-and-whisker plots were used to show the r_*s*_ distributions grouped by lineage (Fig. 4b). The colorectal (median r_*s*_ = 0.59), breast (median r_*s*_ = 0.56) and ovarian (median r_*s*_ = 0.58) cell lines had the highest number of replicates and seemed to display on average the highest level of correlation, albeit all other lineages showed very similar values. For example kidney (median r_*s*_ = 0.52), blood (median r_*s*_ = 0.54) and brain (median r_*s*_ = 0.55) cell lines showed the lowest level of correlation (Fig. 4b). Albeit small, these differences could have arisen due to random effects or to intrinsic biological factors that are different between the lineage groups. To assess which was the case, we performed a two-way ANOVA statistical test, followed by a Tukey’s Honestly Significant Difference post-hoc test for pairwise comparisons. We found that there was a statistically significant difference across lineage groups (model p-value « 0.01) but that was also confounded with the study (model p-value « 0.01).

Taken together, these results reinforce previous findings where a variable level of agreement between the steady state transcript and protein abundances was detected. This also highlights the importance of obtaining protein-level abundance measurements in order to gain further insights into a broad range of biological processes.

### Across sample mRNA-protein expression correlation

**Fig. 5:**
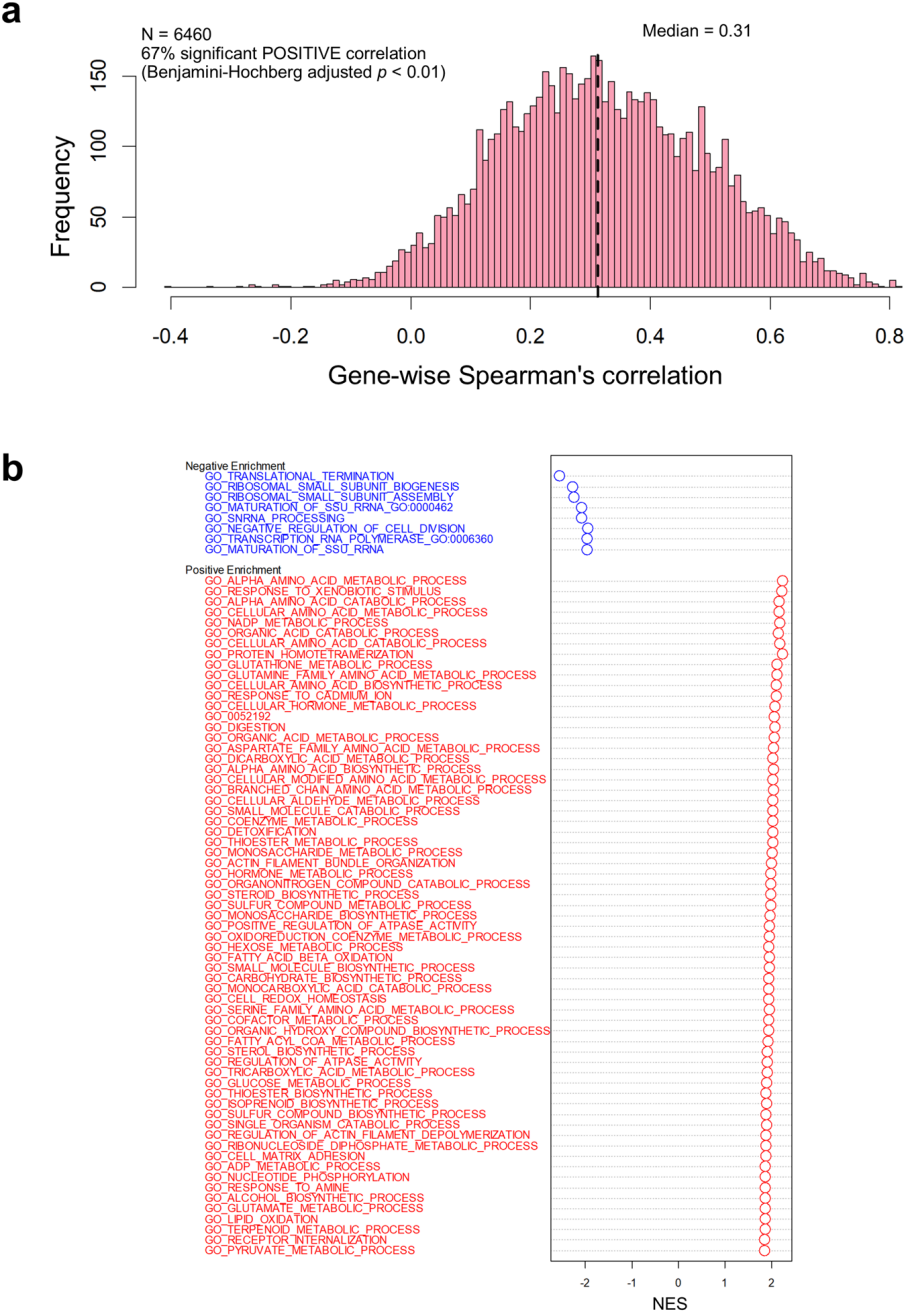
Gene-wise correlation between mRNA and protein levels across all cell lines. (**a**) Histogram of correlation for 6,667 gene-protein pairs across 134 cell lines. (**b**) GSEA showing the top three sets of GO terms significantly represented among those mRNA-protein pairs with highest (red) and lowest correlation (blue) values across the 134 cell lines. Vertical lines indicate the position of the gene set members in the rank ordered list. FDR: False Discovery Rate-corrected p-value.

We investigated the extent of the overall RNA-protein expression correlation across samples. Such analysis consisted on studying how the variation of each transcript and protein originating from the same gene is correlated across all the cell lines. This provided information about whether changes in mRNA levels resulted in abundance changes of the corresponding proteins. An across-sample correlation for a total of 6,667 genes was calculated, of which 4,460 had statistically significant r_s_ values (Benjamini-Hochberg adjusted p-value < 0.01). The median gene-wise r_s_ value was 0.31 ranging from −0.40 to 0.82 (Fig. 5a). Interestingly, negative correlation values were found only for 2% (160) of the mRNA-protein pairs. However, none of those were significant at a 1% FDR level (Benjamini-Hochberg adjusted p-value). In contrast, 16% (1,065) of the genes had a statistically significant correlation that was above 0.5.

Next, the amount of protein variation across cell lines was estimated, by calculating the median abundance and the coefficient of variation for each individual protein. We found that proteins with either high or low variation levels were equally likely to display a high correlation between the mRNA and protein abundance levels. However, the most abundant proteins tended to display higher correlations. A possible explanation is that MS experiments are usually biased towards the most abundant proteins, and therefore more accurate measurements of these proteins are obtained.

Additionally, we studied the level of concordance between mRNA and protein variation considering the biological function. GSEA^42,43^ performed on the list of genes ranked by r_s_, revealed that 65 diverse GO terms were significantly overrepresented among the most correlated mRNA-protein pairs (FDR q-value < 0.01). A similar result, where multiple processes were enriched at the top of the ranked list, was also reported for colorectal cancer^18^. The GO terms sets with a largest NES included *protein homotetramerization* (GO:0051289), *alpha-amino acid metabolic process* (GO:1901605), and *response to xenobiotic stimulus* (GO:0009410) (Fig. 5b). In contrast, only eight GO term sets, related to the ribosome complex, were overrepresented among the least correlated mRNA-protein pairs. The three sets with lowest NES were: *translational termination* (GO:0006415), *ribosomal small subunit biogenesis* (GO:0042274) and *ribosomal small subunit assembly* (GO:0000028) (Fig. 5b).

## Discussion

Recent large-scale genomics and transcriptomics studies have characterized the molecular diversity of cancer to a great depth. However, in order to understand the relationship between genome and disease phenotypes, information about protein expression is increasingly relevant. Since it is difficult for a single study to cover all proteins of interest and/or to capture diverse biological conditions (such as different tumour types or disease stages), meta-analysis approaches enable the computational integration of multiple studies to provide a combined wide-ranging view.

This study provides a rich resource including an aggregated view of protein expression in cell lines and tumour samples, provided to the scientific community through two popular resources: the PRIDE database and EA. Cell lines have indeed provided valuable insights into molecular mechanisms involved in cancer and are generally well-accepted as models of tumour biology. This is possible in part due to the high concordance between molecular signatures, such as RNA expression or single-nucleotide polymorphism (SNP) patterns, which are present both in cell lines and in the tumours of the corresponding lineage^44^. Available studies show different levels of agreement over this statement, and in fact only partial concordance has been found for some of these features^45–47^. Nevertheless, few studies so far have been performed to explore the level of similarity between cell lines and tumours at a proteome-wide level.

We have found that the entire proteomes of breast, colorectal and ovarian cell lines generally mirrored those coming from the tumours. This was indicated by the relatively high level of correlation between baseline protein expression profiles. In fact, only a few cell lines displayed low expression correlations.

It should be pointed out that there are some inherent technical limitations when performing meta-analysis studies like this one. First, although we have used similar quantitative proteomics datasets (only MS1-based quantification approaches performed in Thermo Fischer Scientific Orbitrap instruments), the original data was acquired in different labs in different experimental environments. This inevitably results in the presence of batch effects. We attempted to remove these and to validate the overall methodology. Additionally, it has been shown that different batches from the same cell line type can have a higher degree of heterogeneity than what has been generally assumed, as demonstrated for HeLa cells^48^. Furthermore, it is known that tumours and cancer cell lines harbour multiple genomic alterations, such as gene fusions or splice variants, which could produce alternative protein sequences. Mutations (e.g. SNPs) can also be acquired as the result of consecutive cell culturing^2,48^. Additionally the MS/MS analysis search strategy used in this study focused only on detecting known coding protein sequences, using the UniProt reference proteome, in the same way as performed in all the original studies. Indeed, cell line-specific genome or transcriptome sequences were not available. Therefore, it was not possible to detect any DNA/RNA sequence changes that could manifest at the protein level. However, the effect of this limitation in the analysis should be small. For instance, in a recent comprehensive study comprising different human tissues^49^, the number of variant peptide sequences detected using matching exome data in the analysis was only 2.4% (238 out of 9,848 possible amino acid variants).

When examining peptides detected in tumours that were not present in any of the cell lines, we detected signatures enriched in receptor activity regulators as well as in keratinization. Cell lines are purer than tumour samples, which tend to be contaminated with stromal cells. Although, we made every effort to remove common contaminants from the analysis (keratin and others), the ‘tumour-specific’ proteins detected might not necessarily reflect endogenous tumour biology. These proteins might have been detected due to tumour immune infiltration^50^, contamination from sample processing and/or contamination from surrounding tissues. Altogether, this highlights some limitations of using *in vitro* cultured cells. While many aspects of protein expression in cancer can be studied using cell lines alone, others (for example, as suggested by our analysis, related to regulation of signalling receptor activity) will likely require better models that are able to model tissue architecture and cell-cell interactions, such as organoids^51^.

We expect that analogous meta-analysis studies of proteomics datasets will become increasingly popular, due to the unprecedented growth rate of proteomics datasets in the public domain^24^. In addition to the concrete limitations mentioned above for this study, there are other more generally applied ones that should be mentioned. First of all, manual curation and annotation of public datasets is an essential step. The current level of annotation for public proteomics datasets is lower when compared with transcriptomics studies. Second, the current most accepted analysis software and methodology require a lot of computation resources in the case of meta-analyses, since all data should ideally be analysed together as a whole. This is not always possible due to the overall size of the aggregated dataset. For practical reasons, here we decided to perform the analysis in two batches (cell-lines and tumours), and combine the results on the peptide level or map protein groups to genes and then combine. In case where it was not feasible we used razor proteins in each group to map between the two datasets.

The availability of these results in widely-used resources such as PRIDE and especially in this case Expression Atlas represent, in our view, the right route for proteomics data to be more accessed and consumed by scientists who are non-expert in proteomics.

## Methods

### Data sources and curation

Proteomics data from 11 studies (Online-only Table 1) was collected from public repositories: PRIDE (https://www.ebi.ac.uk/pride/archive/), MassIVE (https://massive.ucsd.edu/), and CPTAC data portal (https://cptac-data-portal.georgetown.edu/cptacPublic/). Raw proteomics data was manually curated to extract processing parameters, experimental design and sample characteristics. The biological metadata was captured in a Sample and Data Relationship Format (SDRF)^52^ before it was loaded into EA as 11 separate tracks, one for each study.

The proteomics studies were selected since they all employed a similar MS platform (Thermo Fisher Scientific Orbitrap) and the resulting protein quantification could be based on the intensity of the peptide precursor ions (MS1 based quantification^53^). Initially we identified many studies, but narrowed down our selection to studies where we had both access to raw data and appropriate annotations (i.e. technical metadata including acquisition mode, processing parameters etc., as well as biological metadata such as sample type, lineage, biological replicates, etc). We did not consider smaller studies where only one cell line (or a small number of tumours) were analysed. The final set of samples was assembled in September 2018 and is summarised in Online-only Table 1.

Transcriptomics data was obtained from EA (https://www.ebi.ac.uk/gxa/home). The following three RNA-seq experiments, designated as ‘baseline’ in EA, were used in this study: E-MTAB-2706, E-MTAB-2770 and E-MTAB-3983. Transcriptomics data have been previously curated in EA, and the biological metadata captured in SDRF format, in a consistent manner with the proteomics data.

### Proteomics raw data processing

Raw LC-MS data was processed using the MaxQuant software. The MS/MS data was searched in two batches (cell line and tumour data separately) against the UniProt human reference proteome (containing canonical and isoform sequences, download date 31.08.2017, 71,591 sequences) appended with sequences of common contaminants provided by MaxQuant. Search parameters were chosen to reflect those used in the original publications. In all cases, carbamidomethylation of cysteine was set as fixed modification and oxidation of methionine and N-terminal acetylation were set as variable modifications. For studies that used SILAC labelling, appropriate SILAC settings were selected. Enzyme specificity was set to trypsin, LysC, chymotrypsin or GluC (according to the enzymes used in the original study), allowing a maximum of two missed cleavages. MS1 tolerance was set to 10 ppm and MS2 tolerance to 20 ppm for FTMS data and 0.4 Da for ITMS data. PSM (Peptide Spectrum Match), peptide and protein identification FDR was set at 1% at each level. All of the processing parameters are available in the *mqpar-celllines.xml* and *mqpar-tumours.xml* files included in the PRIDE PXD013455 dataset.

### Transcriptomics raw data processing

Transcriptomics data stored in EA was previously processed using a standardized pipeline^36^. Briefly, the sequencing reads were quality filtered, which involved the removal of adaptor sequences (adaptor trimming), low-quality reads, uncalled bases (e.g. N) and reads arising from bacterial contamination. TopHat2^54^ was used to perform genomics alignment using the reference Ensembl genome (Ensembl release 79). Default TopHat2 parameters were used. The number of reads that mapped to a particular gene (raw counts) were obtained with HTSeq^55^ and normalised gene abundance was calculated as FPKM (fragments per kilobase of exon model per million reads mapped) values^56^. EA data matrices can be downloaded in a tab-delimited format from the corresponding dataset entry. Importantly, for each dataset, technical and biological replicates were averaged and quantile normalised within each set of biological replicates using the limma package^57^. Finally, in cases where a cell line had replicate measurements across datasets, the average FPKM abundance was used.

### Selection of protein quantification values

Since quantitative proteomics data originating from different studies is heterogeneous and likely to contain batch effects, we developed and benchmarked a procedure to integrate the quantification results. The procedure is described below.

iBAQ protein quantification values were obtained from the corresponding MQ *proteinGroups.txt* files. In some studies, multiple digestion enzymes were employed to characterize the same sample, for example in the dataset from ref. ^10^ (see Online-only Table 1), where all cell line samples were digested with both trypsin and LysC. Because iBAQ quantification takes into account all theoretically observable peptides in a given protein, and these will differ depending on the proteolytic enzyme used, the quantitative analysis was limited to tryptic-digested samples only.

### Proteomics data normalisation

Cell line-derived quantitative data was used to develop and benchmark a normalisation procedure. This was possible because six cell lines (A549, HCT116, HT29, MCF7, RKO and SW620) in the aggregated dataset were acquired in at least three independent studies. It was then assumed that the highest amount of variability in protein expression in those cell lines, and indeed in any other sample type, should arise due to biological differences (i.e. different sample origin), rather than due to technical artefacts, such as the study of origin.

**Fig. 6:**
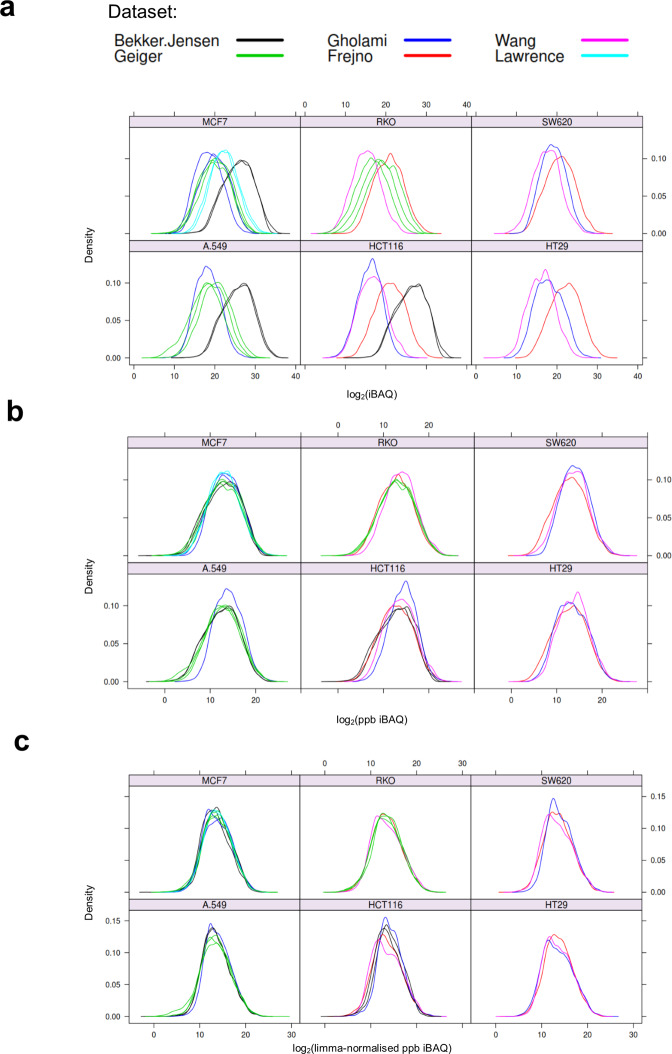
Density plots showing the distributions of log2 transformed iBAQ intensities in six cell lines acquired in at least three studies. Density distribution plots of protein expression values in six cell lines for which biological replicates (in a minimum of three independent studies) were available. Panel (**a**) shows the un-normalised iBAQ values. Panel (**b**) shows the ‘ppb’ normalised iBAQ values. Panel (**c**) shows the final quantification values after applying missing data imputation and batch effects removal with limma. Different colours indicate the study of origin. The plots reveal that strong global effects are present due to the study of origin.

First, the presence of batch effects in the reprocessed data was evaluated by plotting density distributions of log2 transformed iBAQ intensities (Fig. 6a). Upon visual examination of the plots, it was evident that global biases were present between different studies. To correct for these, a two-step normalisation process was applied. As a first step, the individual iBAQ intensities were transformed to “parts per billion” (ppb) for each of the MS runs. Each protein iBAQ intensity value was scaled to the total amount of signal in a given MS run and transformed to ppb, as expressed in the following equation:

$$ppb\_iBA{Q}_{i}=\left(iBA{Q}_{i}/{\sum }_{i=1}^{n}iBA{Q}_{i}\right)\times \mathrm{100,000,000}$$

**Fig. 7:**
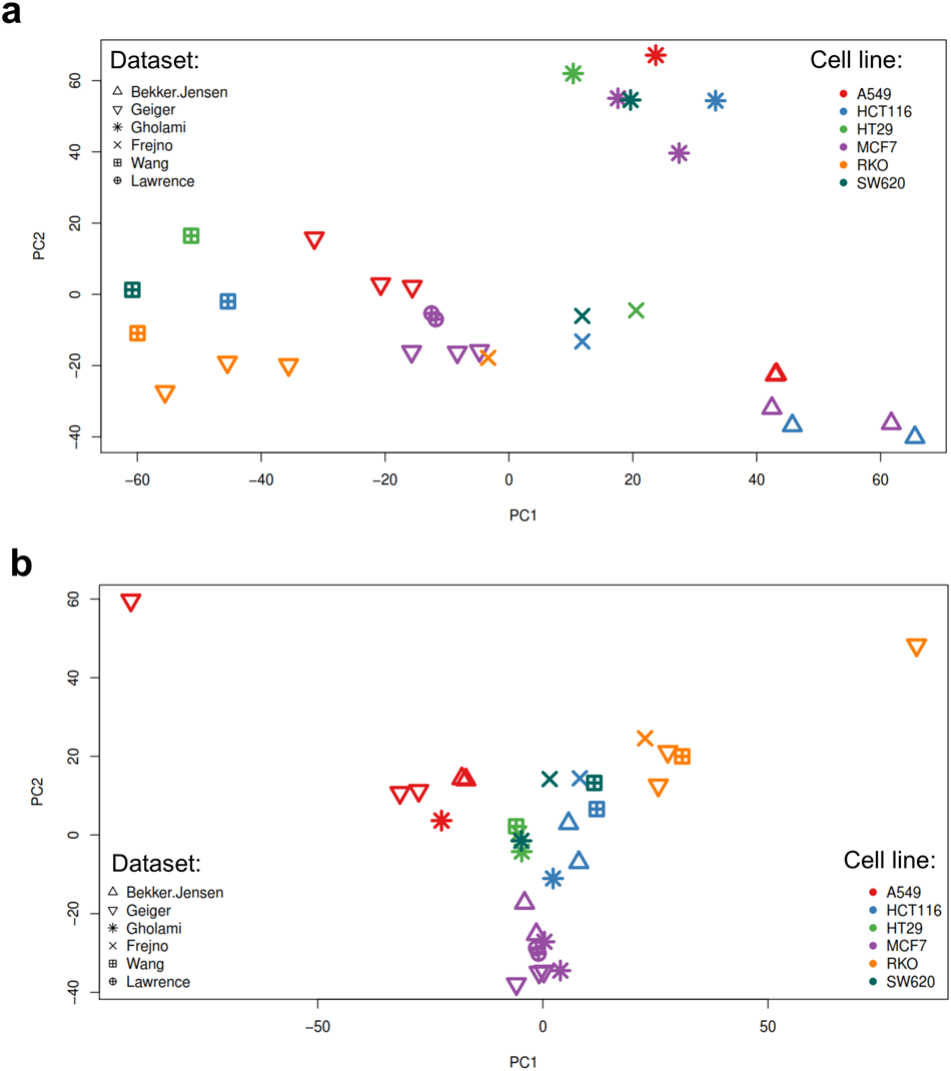
Principal component analysis. A complete matrix across six cell lines (A549, HCT116, HT29, MCF7, RKO and SW620) was used for PCA analysis. This included 2,914 protein expression values. The first two principal components explaining 35% and 30% of the variance are displayed. The colour of the points indicates the sample types whereas the shape of the point indicates the corresponding study. Panel a) shows the PCA space after “ppb” normalisation. It can be observed that points (individual samples) cluster according to the study of origin. Panel b) shows the PCA space after limma batch effects removal. It can be observed that cell lines cluster together based on the lineage rather than based on the study of origin.

As seen in Fig. 6b this procedure mostly removed global differences in the distribution of protein abundances between the studies, which can occur due to different amounts of protein loaded on the chromatographic column, or simply because different MS instruments record data using different numerical scales, among other reasons. However, examination of a principal component analysis (PCA) plot, based on 2,914 proteins quantified in all of the six cell lines, suggested that this simple scaling procedure did not remove the main batch effects due to the study of origin (Fig. 7a).

The cell line and tumour datasets were then filtered to include only proteins that were present in at least 50% of all assays (MS runs). This resulted in the cell line dataset containing 6,514 proteins and a 14% of missing values, and the tumour dataset containing 5,363 proteins and a 16% of missing values. Missing values were then imputed using the conventional Singular Value Decomposition method implemented in the pcaMethods R package (“SVDimpute” function^58^). Finally, the main batch effects were removed separately for each dataset (“cell lines” and “tumours”) using the limma R package^57^ including the study of origin as a covariate (“removeBatchEffect” function). Post-normalisation PCA analysis (Fig. 7b) and inspection of density distribution plots (Fig. 6c) confirmed that the large batch effects among studies of different origin were removed. In the last step, the two datasets were merged by cross-referencing the leading razor protein identifiers.

It must be emphasised that it was only possible to correct for batch effects within each individual “cell lines” or “tumours” protein expression matrix. That is because in many cases, measurements from the same biological sample (i.e. distinct cell line or same tumour type) were acquired in multiple batches (i.e. studies). However, very few overlapping samples were acquired among the “cell lines” and “tumours” datasets.

### Validation of the proteomics data normalisation procedure

In order to further benchmark and validate the normalization procedure it should have ideally been applied to a separate set of previously published datasets and/or to a different set of tumour  samples. However, as we have covered most relevant studies, other datasets where we could readily have applied this strategy were not easily available.

**Fig. 8:**
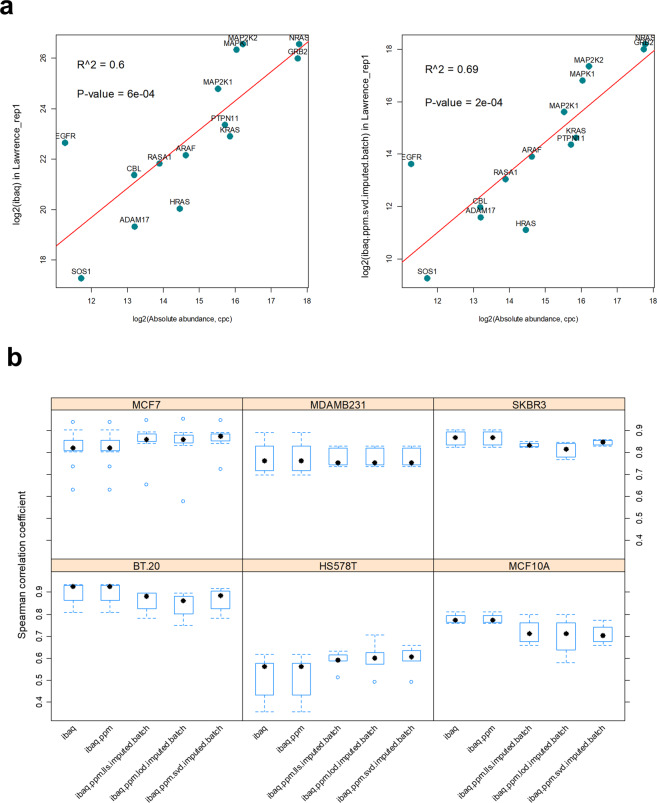
Validation of the normalization procedure by comparison to absolute protein abundances in six cell lines. Panel (**a**) Examplary relationship between estimated absolute protein abundances in copies per cell (cpc) and ibaq or ibaq.ppm.svd.imputed.batch quantification values in the MCF7 cell line. Panel (**b**) Distributions of the calculated Spearman’s rank correlation coefficients between absolute protein abundances and quantification values based on various normalization and missing data imputation methods.

We therefore decided to use an orthogonal approach to validate our method. A common strategy in similar cases is to compare the output of missing data imputation and the normalisation procedure to the known absolute concentration coming from spiked-in peptides/proteins in a given sample, or if not available, from a few “marker” proteins with known abundance. In this case there were no spiked-in standards in the studies that were  reanalysed. Therefore, instead we used absolute abundance estimates coming from a separate study by Shi *et al*.^59^. The authors measured absolute abundance of 25 proteins (14 of those present in our data) in 6 cancer cell lines that were present in our aggregated dataset. The cell lines were BT.20, HS578T, MCF10A, MCF7, MDAMB231 and SKBR3. The 25 proteins were from the EGFR-MAPK pathway and covered a wide dynamic range of 10^2^–10^6^ copies per cell. We compared the raw protein abundance measurements (un-normalised, as extracted from individual studies) and the  final normalized protein abundances (i.e. log2-transformed batch normalized parts per billion (ppb) iBAQ Intensities) to the estimated absolute protein abundances in each cell line, by calculating pairwise Spearman’s rank correlation coefficients (r_*s*_) between all relevant assays. An example of such correlation, before and after normalization, for one assay of MCF7 cell line from Lawrence_CellReports_2015 study, is displayed in Fig. 8a. In addition we made comparisons between iBAQ-based quantification (*ibaq*), iBAQ ppb-normalized (*ibaq.ppm*) and iBAQ ppb-normalized with various missing data imputations and limma-removed batch effect values. Here we benchmarked Limit Of Detection (LOD) imputation (*ibaq.ppm.lod.imputed.batch*), Local Least Squares (LLS) imputation (*ibaq.ppm.lls.imputed.batch*), and Singular Value Decomposition imputation (*ibaq.ppm.svd.imputed.batch* - the method we used in our aggregated dataset). The imputation methods were implemented using the pcaMethods R package^5^. These results are summarised in Fig. 8b. We found that the quantification signal was generally proportional to the actual protein abundance in the cell lines, even for the raw *ibaq* intensities. The median correlation across all cell lines was 0.815 for *ibaq* raw values and improved only slightly for the *ibaq.ppm.svd.imputed.batch* quantification to 0.8286 (Fig. 8b). Importantly, all of the linear models fitted had a p-value < 0.05 indicating that although the correlation was not perfect it was unlikely this relationship was random. Within each cell line, we also found the normalised values were mostly consistent with the absolute protein abundances reported by Shi *et al*. An exception was  the HS578T cell line, which showed poor correlation values (mean r_*s*_ = 0.5), but it improved slightly after our normalization procedure (mean r_*s*_ = 0.6). In addition, the differences between missing data imputation methods were minimal due to the fact that most values were already present in our filtered quantification matrix. In summary, this ‘quality control’ check showed that this procedure was robust and did not bias the final quantification values.

## Supplementary information

Supplementary Table 1

## Data Availability

All the results of this study have been made available via the PRIDE database (dataset PXD013455 - https://www.ebi.ac.uk/pride/archive/projects/PXD013455)^60^. Due to the overall size of the data, the processing was done in two batches denoted as “cell lines” and “tumours” analysis. PRIDE dataset PXD013455 therefore contains raw data files from each study in a corresponding.zip folder, intermediate MQ files (combined-tumours.zip and combined-celllines.zip), and the.txt results files (txt-tumours.zip and txt-celllines.zip) used to create the integrated map of protein expression. In addition normalized protein expression matrices corresponding to each reanalysed study have been included in Expression Atlas (E-PROT-19, E-PROT-28, E-PROT-24, E-PROT-20, E-PROT-25, E-PROT-21, E-PROT-22, E-PROT-26, E-PROT-27, E-PROT-18, and E-PROT-23). The reanalysed public proteomics datasets are indicated in Online-only Table 1. The re-used gene expression values coming from cell lines are available in Expression Atlas (accession numbers E-MTAB-2706, E-MTAB-2770 and E-MTAB-3983).

## Online-only Table

**Online-only Table 1:** List of the public datasets used in this study.

Dataset name and reference	Original Dataset identifier	Sample types	No of MS/MS spectra	No of unique peptides contributed in the aggregated dataset	Expression Atlas Accession Number (protein expression data)
Bekker-Jensen_CellSystems_2017 (ref. ^17^)	PXD004452	Cell lines, 5 common cell lines	1.53 E + 07	2.54 E + 05	E-PROT-19
Coscia_NComms_2016 (ref. ^12^)	PXD003668	Cell lines, ovarian cancer	1.03 E + 07	1.65 E + 04	E-PROT-28
Frejno_MolSysBiol_2017 (ref. ^13^)	PXD005354, PXD005355	Cell lines, colorectal cancer	2.14 E + 07	1.59 E + 04	E-PROT-24
Geiger_MCP_2012 (ref. ^14^)	PXD002395	Cell lines, 11 common cell lines	6.38 E + 06	5.51 E + 03	E-PROT-20
Gholami_CellReports_2013 (ref. ^15^)	PXD005940, PXD005942, PXD005946	Cell lines, NCI-60 panel	1.52 E + 07	1.12 E + 04	E-PROT-25
Iglesias-Gato_EurUrology_2016 (ref. ^20^)	PXD003636, PXD003430, PXD003452, PXD003515, PXD004132, PXD003615, PXD004159	Tumours, prostate	1.38 E + 07	6.26 E + 03	E-PROT-21
Lawrence_CellReports_2015 (ref. ^10^)	PXD008222	Cell lines, triple-negative breast cancer	1.95 E + 07	4.65 E + 04	E-PROT-22
Pozniak_CellSystems_2016 (ref. ^21^)	PXD000815	Tumours, breast cancer	3.43 E + 07	5.34 E + 03	E-PROT-26
Tyanova_NComms_2016 (ref. ^22^)	PXD002619	Tumours, breast cancer	1.84 E + 07	9.62 E + 03	E-PROT-27
Wang_Gastroenterology_2017 (ref. ^9^)	MSV000080374 (MassIVE)	Cell lines, colorectal cancer	5.50 E + 06	1.60 E + 03	E-PROT-18
Zhang_Nature_2014 (ref. ^18^)	https://cptac-data-portal.georgetown.edu/cptac/s/S022	Tumours, colorectal cancer	1.29 E + 07	2.80 E + 03	E-PROT-23

Most of the datasets were obtained from the PRIDE database^24^. The remaining two datasets were obtained from MassIVE^26^ and the CPTAC data portal. The mapping between sample names and their lineage/cancer type and study of origin are listed in Supplementary Table 1.

## Code availability

The scripts used to generate the final quantification values (and selected intermediate files) are available at: https://github.com/J-Andy/Protein-expression-in-human-cancer.
